# Diagnostic and Neurological Overview of Brain Tuberculomas: A Review of Literature

**DOI:** 10.7759/cureus.20133

**Published:** 2021-12-03

**Authors:** Carlos David Perez-Malagon, Raul Barrera-Rodriguez, Miguel A Lopez-Gonzalez, Luis F Alva-Lopez

**Affiliations:** 1 Centro de Ciencias Biomedicas, Universidad Autonoma de Aguascalientes, Aguascalientes, MEX; 2 Immunopharmacology, Instituto Nacional de Enfermedades Respiratorias (INER), Mexico City, MEX; 3 Neurosurgery, Loma Linda University Medical Center, Loma Linda, USA; 4 Radiology, Medica Sur, Mexico City, MEX

**Keywords:** brain surgery, treatment, tuberculosis, brain, tuberculoma

## Abstract

Tuberculosis is a disease caused by a bacteria named Mycobacterium tuberculosis (M. tb). It is estimated by World Health Organization (WHO) that nearly a quarter of the world’s population is infected. Tuberculoma of the brain is one of the most severe extrapulmonary forms that affects patients younger than 40 years of age. Brain parenchymal tuberculoma develops in nearly one of 300 non-treated cases of pulmonary tuberculosis cases. In endemic regions, tuberculomas account for as many as 50% of all intracranial masses. Tuberculoma results in a hematogenous spread of M. tb from an extracranial source. Tuberculomas can mimic a variety of diseases and can present themselves in a subacute or chronic course, from asymptomatic to severe intracranial hypertension. Diagnosis is based on computed tomography (CT) scan and magnetic resonance imaging (MRI) studies with a similar ring-enhancing lesion. Treatment is primarily medical, and the duration for brain tuberculoma can vary from six to 36 months. In certain cases, surgery is recommended.

## Introduction and background

Tuberculosis is one of the oldest infectious diseases of human history caused by Mycobacterium tuberculosis (M. tb). Even if recognized as one of the oldest infectious diseases, it still affects a large amount of the world population, more common in developing countries with the potential to cause disease in almost every tissue of the body, including central nervous system which is one of the most severe forms.

The objective of this manuscript is to review pathophysiology, diagnosis, clinical features, diagnosis, and treatment of brain tuberculoma, which is a rare entity and is associated with high morbidity and mortality.

## Review

Mycobacterium tuberculosis complex is composed by different species, which include the Mycobacterium tuberculosis (M. tb) (that exclusively impacts humans), Mycobacterium canettii, Mycobacterium africanum (isolated from African population), a wider host spectrum Mycobacterium bovis (affects humans, bovines and goats), Mycobacterium microti (rodent pathogen), Mycobacterium caprae (only affects goats) and Mycobacterium pinnipedii (affects seals only), which are all genetically related. [1] All these mycobacteria share 99.9% similarity at the nucleotide level, with an identical 16S rRNA sequences [2,3]; however, they differ in their host tropisms, phenotypes, and pathogenicity [4].

Epidemiology

Tuberculosis (TB) has been a significant public health problem in both developing and developed countries. However, it is an illness surrounded by poverty, economic distress, vulnerability, marginalization, and causes stigma and discrimination among affected people [5]. 

The latest WHO report estimates that nearly a quarter of the world’s population is infected with M. tb [5].

TB remains one of the leading causes of death by a single infectious organism. Fortunately, it is no longer included in the global top 10 diseases, falling from 7th to 13th place in the last decade. The annual number of TB deaths is lowering globally; yet the pace is not fast enough to reach the first milestone of the End TB Strategy, i.e., a 35% reduction in active cases of TB between 2015 and 2020.

In 2019, about 10 million people developed TB and 1.4 million people lost their lives, including 208 000 HIV-positive patients. Tuberculosis affects adults in about 90% of the cases, with men being more affected than women.

Pulmonary TB is the most common clinical presentation according to previously published data (85.67% vs. 14.75% extrapulmonary). [6] Among extrapulmonary tuberculosis (ExTB), central nervous system (CNS) is one of its most aggressive manifestations. CNS occupies the sixth to seventh more common tissues affected (2%-15%) among ExTB, reaching the highest in subjects with acquired immunodeficiency syndrome (AIDS) [7-11]. In contrast to the pulmonary form, females are more affected by CNS TB and the patients are more likely to be young (<40 years old). Furthermore, 60%-70% of cases are younger than 20 years old, with cases surging in the first five years of age, although rare in children under three months [12-14].

Most patients with CNS TB have different causes of immune suppression, such as HIV, diabetes mellitus, malnutrition, chronic renal failure, cancer, use of chemotherapy or other immunosuppressive drugs, etc. [1].

Mortality due to CNS disease, mainly related to TB meningitis, is seen in children under 13 years at 42.2%, and 2.7% in adults. [15] In autopsies, meningeal involvement in TB patients was 19.3% in subjects younger than 20 years old and 5.9% in those above 20 years old [7]. Outcome in patients with CNS TB tends to be adverse but seems to be higher in those with meningitis [14].

Brain parenchymal tuberculoma

It is estimated that TB in the brain parenchyma develops in nearly one of 300 non-treated cases of pulmonary TB cases, and in half of the patients with disseminated TB. Several studies have described that almost 75% of the patients had developed pulmonary TB six to 12 months before diagnosis of CNS TB [16-18]; however, around 25% to 30% of patients with brain TB are not affected by pulmonary TB. 

In regions where TB is an endemic disease, tuberculomas account for as many as 50% of all intracranial masses [19].

Pathophysiology

M. tb is an airborne disease that spreads from one person to another in infectious droplets [0.65 (small) to > 7.0 μm (medium-large)] [20] through coughing, sneezing or talking. Larger droplets are trapped by mucus in the upper airways or oropharynx, where these particles can produce TB of the oropharynx or cervical lymph nodes [20,21]. In contrast, the smallest particles travel from the nasopharyngeal to tracheobronchial region and deposited in the distal airways up to alveoli.

Mycobacteria deposited in the respiratory alveoli are phagocytosed by alveolar macrophages, and with the help of dendritic cells, alveolar neutrophils also act against M. tb [22,23]. Once mycobacteria are engulfed by macrophages, endocytic compartments convert to phagosomes, which fuse into lysosomes. The phagosomal contents are then presented to different substances (reactive oxygen, lysosomal hydrolases, and nitrogen products) that help to end the mycobacteria that reside within immune cells. This inflammatory response attracts more immune cells to the site of infection, leading to the formation of granulomas, which serves to limit the infection and the damage to surrounding tissue that restricts the dissemination of M. tb to other organs [24,25]. However, if M. tb is not controlled, dissemination initiates in lymphatics and lymph nodes. During all these processes, mycobacteria try to avoid immune response of the host to continue with their tissue invasion [26].

When the granuloma restricts the infection, mycobacteria become dormant (latent tuberculosis) and may live for years without producing clinical disease. However, at any moment, dormant bacilli can reactivate and cause disease in the lungs or disseminates through lymphatics or blood vessels to any other tissue. This usually occurs if the host has any condition that causes depression of the immune system [1,25]. In this sense, previous studies have observed that in mice that lack microfold cells, dissemination of M. tb to lymph nodes is reduced, and it has been suggested that the presence of microfold cells in the host might favors the development of ExTB [27].

TB of the central nervous system results from hematogenous dissemination of M. tb from an extracranial source [28]. Intracranial manifestations of TB are wide and can affect different anatomic sites [12,29], such as meningeal or parenchymal, and by type such as diffuse meningitis, tuberculoma, tuberculous abscess, focal cerebritis, vasculitis and strokes (23%). However, tuberculoma (granuloma) and tuberculous meningoencephalitis are the two most important manifestations of tuberculosis of the CNS, thus, an early diagnosis and treatment are crucial for effective treatment [30].

Theoretically, M. tb is capable to surpass the blood-brain barrier (BBB) as a free (extra-cellular) organism or via monocytes/neutrophils with the bacilli [31,32].

Once the bacilli have reached the brain, several cytokines (TNFα, IL-1, and IL-6) are produced by various cells, such as macrophages, microglia, and astrocytes that increase the permeability of endothelial cells [33] penetrating the BBB [31,34,35,36].

Rich and Mc Cordock described that cerebral TB develops in two steps [37]. First, small tuberculous lesions in the brain (Rich’s foci) tend to coalesce and enlarge during the stage of bacteremia. These tubercles can produce central caseous necrosis, epithelioid cells, lymphocytic infiltrates, and giant cells. Second, Rich’s foci can rupture and develop several types of CNS tuberculosis, depending on where they placed their content [29,37,38]. The rupture of these lesions can cause meningitis when the content flows into the subarachnoid space or into the ventricular system [37].

Rich’s foci lesions can also be found in the meninges, the subpial or subependymal surface, and might remain in a dormant manner for long periods of time [37].

In an autopsy series of cases with parenchymal brain tuberculomas, the frontal lobe was slightly more commonly affected in 35.3% of the patients, followed by temporal and parietal lobes at 29.4% each, and at 5.9% for the occipital lobe [39].

Clinical features

Tuberculomas can also mimic other entities, including glioblastoma, brain metastasis, intracranial hemorrhage, abscesses, or other gliomas, which can be associated with calcifications that produce the “target sign” that suggest reactivation or dormant tuberculosis.

Tuberculomas of the brain can manifest in a subacute or chronic illness, lasting from weeks to months, with predominance in immunocompromised patients. If patients develop isolated or scant small parenchymal lesions, clinical course might be asymptomatic, but if these lesions are multiple or large, common symptoms are fever, vomiting, headache, focal neurological deficits, seizures, hydrocephalus, meningeal irritation signs and intracranial hypertension with papilledema [40,41,42].

In patients with HIV, tuberculomas can cavitate and fill with fluid, turning into abscesses. In some cases, abscesses can rupture into the epidural space and through intervertebral foramen, which can cause paraplegia. Abscesses in the brain parenchyma are rare, and treatment is conducted by draining the abscess surgically [7].

Diagnosis

Among TB patients, CNS is affected in 1.8%, and brain tuberculoma is the only manifestation seen in 24% of the cases [14].

It is important to analyze the clinical course and evaluate predisposing factors capable of suppressing the immune system, which favors acquired opportunistic infections. When brain tuberculoma is the only lesion, cerebrospinal fluid (CSF) analysis might be normal; however, due to the increased intracranial pressure, the white count might be elevated favored by the secondary ischemic brain parenchyma. Ziehl-Neelsen staining of cerebrospinal fluid (CSF) for acid-fast bacilli (AFB) (sensitivity 70%, specificity 97.1%) and culture of CSF for M. tb [43,44] are negative when brain parenchyma is involved [45]. PCR for CSF can be useful to diagnose brain tuberculoma; however, it might not be useful for a rapid diagnosis and treatment. [46]

Imaging studies, such as CT and MRI with contrast enhancement, are the basis for diagnosis of tuberculoma [38,42]. The most common image of tuberculoma is a ring-enhancing lesion due to the absence of blood supply in the caseous necrosis center within the tuberculoma [47,48].

However, several diseases are also capable of producing similar imaging features, such as cysticercosis, toxoplasmosis, demyelinating disorders, bacterial abscesses, cryptococcosis, syphilis, sarcoidosis, Behcet disease, radiation encephalopathy, cerebral venous thrombosis, several inflammatory or vascular abnormalities, brain neoplasms (glioblastomas, low-grade gliomas, lymphomas and brain metastases), and unusual cases including free-living amoebas, such as Naegleria, Balamuthia mandrillaris and Acanthamoeba [48,49].

Computed tomography

Selvapandian et al. in India reported 100% sensitivity of CT scan and 86% specificity for diagnosing tuberculoma. However, the positive predictive value can be as low as 33% even in a high-incidence population [50].

**Figure 1:**
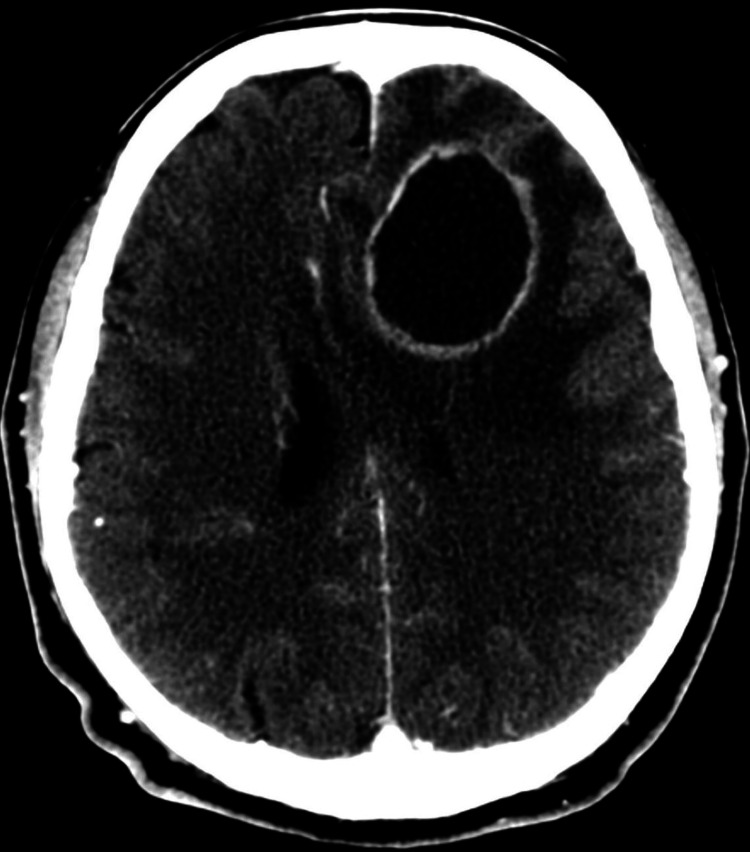
Tuberculoma on head CT scan. Enhanced head CT scan in axial views of an HIV patient with a left frontal tuberculoma showing hypodense necrotic central area with ring enhancement, and perilesional severe edema with subfalcine herniation (Courtesy of Doctor Felipe Alva-Lopez, co-author).

CT images show hypodense or isodense mass lesions on non-contrast studies, but after contrast administration, the ring- or homogeneous disk-like enhancement with an inner region of hypodensity can be observed. The enhancing lesions have perilesional vasogenic edema and are frequently located at the junction of gray and white matter as well as in the sub-cortical area and can be either deep or superficially in the brain parenchyma (Figure 1) [49]. When patients are treated with adequate antituberculosis therapy, serial CT scans might show complete disappearance of these lesions. [51]

Magnetic resonance image

**Figure 2:**
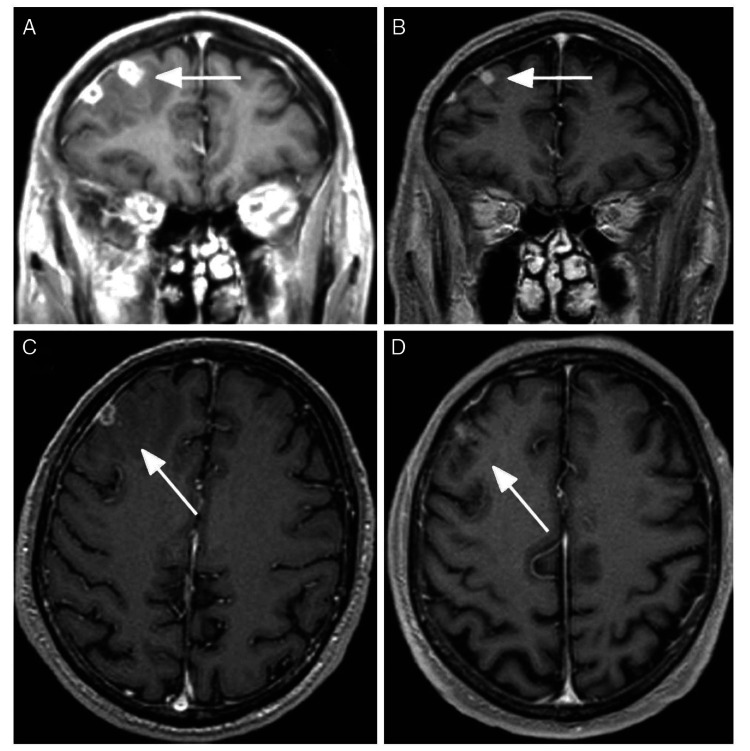
Enhanced brain MRI before and after treatment. Contrast-enhanced brain MRI of patient with tuberculoma in right frontal area with perilesional edema. A. Coronal image before treatment; B. Coronal image three months after treatment showing reduced size of the lesions; C. Axial image before treatment; D. Axial image three months after treatment [Courtesy Bessolo E, Villate S, Arroyo JA, Rango G, Ortiz GA. Tuberculoma cerebral en placa. Presentacion atipica de tuberculosis en el sistema nervioso central. Neurol Arg 2017;9(3):195-198].

MRI is slightly superior for showing the size of brain lesions and helps to identify the solid caseous necrosis [52]. Images of caseating tuberculoma are generally composed of three zones, an inner iso-intense and hypo-intense layer image due to caseous necrosis (signals in T1WI and T2WI, respectively) [28]. In this zone, fluid-attenuated inversion recovery (FLAIR) images reflect extensive necrosis and hypercellularity [53]. A middle layer with hypo-intense and hyperintense signals due to the presence of Langhans giant cells, epithelioid cells, and edema (in T1WI and T2WI, respectively) is enhanced with gadolinium in contrast images, whereas the external layer shows iso-intense and hypo-intense component (signals in T1WI and T2WI) due to the collagenous capsule (Figure 2) [28,53].

.

When patients have a non-caseating tuberculoma, images usually show hyperintensity on T2-WI and slight hypo-intensity on T1-WI [49].

Wasay et al. [53] reported that diagnosis of brain tuberculoma in a group of patients was made with different methods: 19% was based on pathology, 57% on clinical findings or neuroimaging after response to tuberculous chemotherapy, and 24% on evidence of systemic tuberculosis. According to the number of lesions, 31% of cases had a unique lesion, and 69% had multiple lesions. The number of lesions varied from 1 to >100 (mean = 4.5 lesions/patient). After contrast, diameter of tuberculomas ranged from 1 mm to 5 cm [49,53].

It has also been suggested that lesions larger than 1 cm might show enhancement with irregular shapes, ringlike shapes, open rings, and lobular patterns [49]. Other findings can be cortical and subcortical infarcts (12%), edema (33%), calcification (10%), mass effect, and/or midline shift (18%) and meningeal enhancement (12%).

A few, new, promising diagnostic techniques for the diagnosis of brain tuberculoma are being utilized, such as magnetic resonance spectroscopy, diffusion-weighted MRI, positron emission tomography and a promising perfusion-weighted MRI, which might aid in diagnosis. Nevertheless, there is scant experience in patients with brain tuberculoma.

Magnetic resonance spectroscopy (MRS)

MRS has not been used widely and determines the concentration of brain metabolites, such as N-acetyl aspartate, choline, creatine, and lactate in the brain tissue. In brain tuberculoma, this study provides chemical information of major lipid-lactate peaks (usually 86%). This information, in addition to MRI, might increase specificity [54,55,56].

Diffusion-weighted magnetic resonance imaging

This advanced diagnostic tool shows that signal intensities of lesions on diffusion images and the apparent diffusion coefficients (ADC) ranged from 0.406 to 2.64 x 10(-3) mm2/s (mean +/- SD: 1.038 +/- 0.609 mm2/s). Lesions with hyperintense centers on T2-weighted images have increased intensity, and in hypointense centers on T2-WI have decreased signal intensity on diffusion images. Diffusion-weighted MRI and magnetic resonance spectroscopy could be useful in the evaluation of focal cerebral tubercular lesions, such as in brain metastasis and gliomas [57,58].

Positron emission tomography has very low possibilities to distinguish brain tuberculoma from neoplastic lesions [49].

When diagnosis of tuberculoma has not been made with non-invasive methods, or there has not been a positive response after antituberculosis treatment, the definitive diagnosis should be made by a CT-guided biopsy, preferably stereotactic biopsy, in order to minimize tissue injury [49,59,60,61]. However, excision might be necessary [62].

Biopsy also serves in cases where imaging studies show progression of brain lesions, such as a paradoxical response to antituberculosis drug therapy, or when patient is infected with drug-resistant TB, or a non-compliant patient [28,50].

Assefa et al. reported that in 222 operated cases with intracranial masses, histopathological analysis demonstrated that 58 (26.1%) cases were due to tuberculosis. Out of these cases, 29 (12.6%) were non-caseating tuberculomas, 28 (13.1%) were tuberculomas with caseous necrosis and one (0.4%) was a tuberculous abscess [42].

Treatment

The management for brain tuberculomas is mainly pharmacological with different first-line antituberculous drugs. Some authors recommend empirical medical therapy without the need for histological confirmation, while others consider that such therapy should be given until a confirmatory diagnosis is made [28]. The drugs of antitubercular regimen and the duration for treatment for brain tuberculoma is not clearly understood [38].

It has been established that medical treatment for ExTB, including severe forms, is conducted with the current preferred regimen composed of a two-month intensive phase of isoniazid (H), a rifamycin (rifampin [R] or rifabutin), pyrazinamide (Z), and ethambutol (E), followed by a continuation phase of four-month of H plus R or rifabutin [63,64,65].

Although Rajeswari et al. proposed a short-course regimen of nine months' duration as an effective treatment of brain tuberculoma, they observed that at the end of treatment, clinical status of patients was good in more than 88% of the patients, and CT scan lesions disappeared in more than 77% of the sample. Moreover, after treatment, 88% of the patients were clinically normal, and none faced a relapse requiring treatment [66]. Thus, it is important to consider that clinical recovery is faster than CT scan clearance.

Other series suggest that brain tuberculoma should be managed with the longer treatment of up to 12-18 months with antituberculosis treatment, with or without surgery. However, according to Nair et al., some patients may require longer periods of treatment [67]. Other studies suggest antitubercular therapy should be from 12 to 30 months of effective antituberculosis drugs [68].

When there is evidence of a new intracranial tuberculoma, or of an expanding existing lesion, it is not necessary to change the antituberculosis regimen [28,69].

Some factors have been associated with the need for prolonging antitubercular treatment in brain tuberculoma for more than 24 months, such as multiple lesions and tuberculomas larger than 2.5 cm. During treatment, some authors have observed that 78% of brain tuberculomas will resolve within 12-24 months of treatment, whereas 22% required >24 months. Yet, in cases with multiple tuberculomas, a median duration for resolution is 36 months. On occasion, brain tuberculomas might be surgically excised totally or need to be reduced in size to <2.5 cm to enable early resolution [67].

In some patients, despite improvement during the first two weeks of an adequate treatment, brain tuberculous lesions can become larger with worsening of clinical status. This phenomenon is known as “paradoxical response”, which seems to be caused by extreme inflammation induced by excessive release of antigens and proinflammatory cytokines produced by M. tb. [34] In these cases, systemic dexamethasone or TNF antagonists can be added as an adjuvant therapy for four to eight weeks [28,69]. After an appropriate treatment of brain tuberculoma, nearly 50% of patients can recover completely, whereas 10% of patients would not recover and die [68,69].

Some case reports have shown that the use of thalidomide might be helpful when responding to antituberculosis drugs, and a high dose of corticosteroids is not satisfactory [70].

Surgery is mandatory when brain tuberculoma is larger than 20 mm [71,72] or when it produces a mass effect on the brain. An elevated intracranial pressure makes it a surgical emergency as with other space-occupying lesions, or when medical therapy has failed completely [28,38,50,51]. Most important sequels are neurological impairment due to endarteritis, hydrocephalus secondary to obstruction of CSF and lesion to cranial nerves.

## Conclusions

Tuberculosis of the brain is a rare extrapulmonary infectious disease caused by blood dissemination of M. tb in young, immunosuppressed subjects leading to high mortality. Clinical course tends to be from weeks to months and requires prompt diagnosis and treatment. Diagnosis is basically made with CT and MRI and in certain cases brain biopsy is necessary. A four-drug regimen might be sufficient for treating patients, although occasionally, surgical resection can be considered.
